# Allergic sensitization to common pets (cats/dogs) according to different possible modalities of exposure: an Italian Multicenter Study

**DOI:** 10.1186/s12948-018-0081-z

**Published:** 2018-02-02

**Authors:** G. Liccardi, L. Calzetta, G. Baldi, A. Berra, L. Billeri, M. Caminati, P. Capano, E. Carpentieri, A. Ciccarelli, M. A. Crivellaro, M. Cutajar, M. D’Amato, I. Folletti, F. Gani, D. Gargano, D. Giannattasio, M. Giovannini, C. Lombardi, M. Lo Schiavo, F. Madonna, M. Maniscalco, A. Meriggi, C. Micucci, M. Milanese, C. Montera, G. Paolocci, R. Parente, A. Pedicini, R. Pio, F. Puggioni, M. Russo, A. Salzillo, P. Scavalli, N. Scichilone, B. Sposato, A. Stanziola, G. Steinhilber, A. Vatrella, P. Rogliani, G. Passalacqua, On behalf of Italian Allergic Respiratory Diseases Task Force, Ilaria Baiardini, Caterina Bucca, Giorgio Walter Canonica, Maria Teresa Costantino, Stefano Del Giacco, Enrico Heffler, Stefania La Grutta, Vincenzo Patella, Erminia Ridolo, Giovanni Rolla, Oliviero Rossi, Eleonora Savi, Gianenrico Senna, Carlo Filippo Tesi, Giovanni Viegi

**Affiliations:** 1 Department of Pulmonology, Haematology and Oncology. Division of Pneumology and Allergology, High Speciality “A. Cardarelli” Hospital, Naples, Italy; 2 Postgraduate School of Respiratory Medicine. Department of Experimental Medicine and Surgery, University of Rome “Tor Vergata”, Rome, Italy; 3 Department of Experimental Medicine and Surgery, University of Rome Tor Vergata, Rome, Italy; 4 Respiratory Medicine Unit, ASL (District 66), Salerno, Italy; 5 Respiratory Allergy Unit, G. Da Procida Hospital, Salerno, Italy; 6 Department of Laboratory Medicine, University Hospital Padova, Padua, Italy; 7 Asthma Center and Allergy Unit, Verona University and General Hospital, Verona, Italy; 8 Unit of Pulmonary Immunology and Respiratory Diseases Ospedale “Santa Maria Della Speranza”, Battipaglia, Salerno Italy; 9 Division of Pneumology, “Santa Maria Novella” Hospital, Galatina, Lecce Italy; 10 Allergy Unit, Loreto Crispi Hospital, Naples, Italy; 11 Unit of Allergy and Occupational Medicine, University Hospital Padova, Padua, Italy; 12 Allergy Center, Division of Internal Medicine, Ospedali Riuniti Penisola Sorrentina, Sorrento, Naples, Italy; 13 Department of Respiratory Disease, “Federico II” University – AO “Dei Colli”, Naples, Italy; 14 Department of Medicine, Section of Occupational Medicine, Respiratory Diseases and Toxicology, Terni Hospital, University of Perugia, Perugia, Italy; 15 Allergy Unit, S. Luigi Gonzaga Hospital, Orbassano, Turin, Italy; 16 Allergy Unit, High Speciality “San Giuseppe Moscati” Hospital, Avellino, Italy; 17 Respiratory Physiopathology and Allergy, High Speciality Center, “Mauro Scarlato” Hospital, Scafati, Salerno, Italy; 18 Pulmonary Diseases Department, Mirandola Hospital, Modena, Italy; 19 Departmental Unit of Allergy, Clinical Immunology and Respiratory Diseases, Fondazione Poliambulanza, Brescia, Italy; 20 Allergy and Clinical Immunology, “G. Fucito” Hospital, S. Giovanni di Dio e Ruggi D’Aragona University Hospital, Salerno, Italy; 21 Allergy Unit, ASL (Sanitary District n°12), Caserta, Italy; 22 Pulmonary Rehabilitation Unit, ICS Maugeri, Telese Terme, Benevento Italy; 23 Allergy and Immunology Unit, Fondazione “Salvatore Maugeri”, Institut of Research and Care, Scientific Institute of Pavia, Pavia, Italy; 24 Division of Pneumology and Allergology Hospital “Carlo Urbani”, Jesi, Ancona Italy; 25 Division of Pneumology, S. Corona Hospital, Pietra Ligure, Savona Italy; 26 Division of Allergy and Clinical Immunology, University of Salerno, Salerno, Italy; 27 Division of Internal Medicine and Allergy, Fatebenefratelli Hospital, Benevento, Italy; 28 Respiratory Diseases Department-IRCCS Humanitas Research and Clinical Center, Rozzano, Milan, Italy; 29 Unit of Respiratory Physiopathology, Allergy and Occupational Medicine, ASL Viterbo, Viterbo, Italy; 30 Biomedical Department of Specialistic and Internal Medicine, University of Palermo, Palermo, Italy; 31 Pneumology Unit, Azienda Ospedaliera “Misericordia”, Grosseto, Italy; 32 Division of Pneumology, Spedali Civili Brescia, Brescia, Italy; 33 Department of Medicine and Surgery, University of Salerno, Fisciano, Italy; 34 Allergy and Respiratory Diseases, Policlinico San Martino, University of Genoa, Genoa, Italy

**Keywords:** Allergic rhinitis, Allergic sensitization, Bronchial asthma, Cat, Dog, Pets exposure, Hypersensitivity, Pets

## Abstract

**Background:**

The query “are there animals at home?” is usually administered for collecting information on anamnesis. This modality to consider exposure to pet allergens constitutes a potential bias in epidemiological studies and in clinical practice. The aim of our study was to evaluate/quantify different modalities of exposure to cat/dog in inducing allergic sensitization.

**Methods:**

Thirty Italian Allergy units participated in this study. Each centre was required to collect the data of at least 20 consecutive outpatients sensitized to cat/dog allergens. A standardized form reported all demographic data and a particular attention was paid in relieving possible modalities of exposure to cat/dog.

**Results:**

A total 723 patients sensitized to cat/dog were recorded, 359 (49.65%) reported direct pet contact, 213 patients (29.46%) were pet owners, and 146 subjects (20.19%) were exposed to pets in other settings. Other patients were sensitized by previous pet ownership (150–20.75%) or indirect contact (103–14.25%), in 111 subjects (15.35%) any contact was reported.

**Conclusions:**

Only 213 patients (29.46%) would be classified as “exposed to animals” and 510 (70.54%) as “not exposed” according to usual query. Our classification has shown that many “not-exposed” subjects (399–55.19%) were “really exposed”. The magnitude of exposure to pet allergens at home is not related exclusively to pet ownership. These considerations should be taken into account during the planning of epidemiological studies and in clinical practice for the management of pet allergic individuals.

## Background

Exposure to animal allergens constitutes a relevant risk factor for the development of allergic sensitization and respiratory allergic diseases, such as asthma and rhino-conjunctivitis in susceptible individuals [1]. In all developed countries cats and dogs are the most common pets living in indoor environments and the frequency of their ownership is highly variable, according to cultural differences and environmental factors [2, 3]. Cat and dog allergens should be considered ubiquitous because they are found not only in indoor environments, where these animals are kept, but also in other indoor private or public places where cats/dogs have been never kept [4]. Although the presence of a pet at home is considered usually the main risk factor for allergic sensitization, dynamic distribution of the main pet allergens indoors is complex and depends by production, aero-dispersion, sedimentation and passive transport through clothes and other items [5–9]. These variables determine a diffuse presence of pet allergens (indirect exposure) also in indoor environments without pets and in environments where pets are no longer present for a long time (e.g. voluntary removal or re-location, natural death etc.) [10–12]. The query “are there animals at home?” is common and usually administered by researchers, physicians and pulmonologists/allergologists to patients for collecting information on anamnesis [13]. This prevalent modality to consider exposure to pet allergens constitutes a potential bias in large epidemiological studies on the relationship between pet-exposure and allergic sensitization [14]. We believe that an accurate medical history on pet exposure is essential also in clinical practice for an objective evaluation of the risk and the clinical significance of the skin-prick-test (SPT) positivity to pet (cat/dog) allergens, as well as for the management of sensitized patients (pet-avoidance measures, allergen immunotherapy, pharmacological treatment of respiratory symptoms etc.) [14]. The aim of our study was to evaluate and quantify the role of different modalities of exposure to cat/dog in inducing allergic sensitization in a consistent population of cat and/or dog sensitized individuals living in Italy.

## Methods

**Fig. 1:**
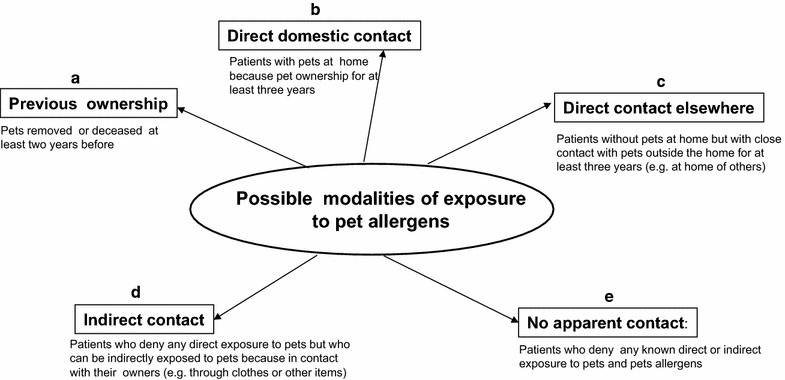
An overview of modalities of exposure to pet allergens reported in our standardized form (Modified from [10])

Thirty Allergy units, distributed over the whole national territory and belonging to the “Italian Allergic Respiratory Diseases Task Force” participated in this cross-sectional study. Each centre collected data of at least 20 consecutive outpatients, referred for actual asthma and/or rhinitis and sensitized to cat/dog allergens. Data were collected from January 1 to June 30 2013. All centres followed the same protocol, recorded the results in a previously agreed form and obtained an informed consent. Subjects with occupational exposure to cat/dog (farmers, stable-men, or veterinary doctors) were not considered to avoid possible inhibition of SPT responses as a consequence of exposure to higher amounts of pet allergens. Patients with chronic infectious diseases, malignancies or dysmetabolic diseases, severe cutaneous disorders, negative skin reaction to histamine, or treated with drugs interfering with the skin response were excluded from the study [15]. The standardized form reported: demographic data, type and duration of respiratory symptoms, results of the SPTs. Since the absence of a pet at home does not exclude a direct exposure to pet outside and the presence of a pet at home must not be considered the only criterion of pet contact, the assessment of further possible modalities of exposure to cat/dog was considered with specific regard (Fig. 1). The forms had to be filled by the allergist, who also verified the consistency of clinical history and SPT results. Then, the same doctor confirmed the diagnosis of respiratory allergy according to the International Guidelines [16, 17]. The commercial allergen extracts used for screening SPTs were provided by ALK Abello Group, Milan Italy. All centres used the same standard panel of allergens including: *Dermatophagoides pteronyssinus*, *Dermatophagoides farinae*, *Alternaria alternata*, *Cladosporium herbarum*, cat dander, dog dander, *Parietaria*, Grass mix, *Artemisia vulgaris*, *Olea europaea*, *Betula pendula*, *Cupressus sempervirens*, and *Corylus avellana*. These allergens covered the majority of allergens causing respiratory allergy in Italy. Positive (10 mg/ml histamine HCl) and negative (saline solution in glycerine-phenol solution) controls were used. SPTs were carried out and interpreted according to international guidelines [18]. The results were read after 15 min and expressed as the mean of the major wheal diameter plus its orthogonal. A skin reaction of 3 mm or greater was considered positive. SPTs were always performed by the same operator in each centre. The profiles of the wheals were outlined using a fine-point marking pen and transferred by adhesive tape onto patient’s form.

## Results

The participating centres were distributed over the Italian territory. A total of 723 patients sensitized to cat/dog as well as to other allergens were registered, and the main characteristics of these subjects are summarized in Table 1. No patients were mono-sensitized to the common pets, the low percentage of pet-mono-sensitized patients has been described also by other authors [19]. Since all cat/dog-sensitized patients showed multiple cutaneous positivity to other common allergens (mites, pollens and moulds), we could not quantify the role of cat/dog sensitization on the entire duration of allergic symptoms during the year. As shown in Table 2, 359 patients (49.65%) reported direct pet contact, 213 patients (29.46%) were pet owners (pets at home), and 146 subjects (20.19%) were directly exposed to pets in other settings (for at least 3 years in both groups). The remaining patients were likely sensitized because previous pet ownership (150–20.75%) or indirect contact through pet-contaminated items (103–14.25%), in 111 individuals (15.35%) any apparent (direct/indirect) contact was reported. In Table 3 the number and % of patients reporting a worsening of respiratory symptoms after direct exposure to pets is shown. Three hundred seventy-five patients (51.8%) reported an evident aggravation of rhino-conjunctivitis/asthma following pet contact, while 10 patients (1.3%) denied any clinical effects. The cat allergy was more common than that to dog in inducing acute respiratory symptoms in susceptible individuals (259 vs. 56 subjects, respectively). Because monoclonal antibody-based methods to measure the amount of cat/dog allergens in the dust of indoor environments were not available in Italy, we have not information about the levels of indoor exposure to pet allergens.

**Table 1:** Characteristics of the patients sensitized to dog/cat allergens (total no. = 723)

	No (%)
Age range (years)	
0–20	202 (28.0)
21–40	318 (44.0)
41–60	159 (22.0)
> 60	44 (6.0)
Sex: male/female	384/339 (53.1/46.8)
Family history of atopy (yes/no)^a^	358/365 (49.5/50.4)
Intermittent/mild persistent asthma^b^	137 (17.2)
Moderate/severe persistent asthma^b^	80 (10.1)
Intermittent/mild persistent rhinitis^b^	274 (34.4)
Moderate/severe persistent rhinitis^b^	305 (38.3)
Allergic sensitization to common pets	
Dog	160 (22.1)
Cat	256 (35.4)
Dog/cat	307 (42.5)
Allergic sensitization only to cat/dog	0 (0)
Age of onset of respiratory symptoms (years)	
0–20	493 (68.2)
21–40	176 (24.3)
41–60	51 (7.1)
> 60	3 (0.4)
Smoking habit	
Never	479 (66.3)
Actual	105 (14.5)
Past	79 (10.9)
Passive smoke only	60 (8.3)
Previous immunotherapy	
No	622 (86.0)
Yes (none for pets)	101 (14.0)

^a^At least one parent with history of asthma/allergic rhinitis/atopic dermatitis/food allergy

^b^Diagnosis of asthma and/or rhinitis (the majority of patients have shown both symptoms)

**Table 2:** Possible modalities of exposure to pet allergens in 723 pet-sensitized patients (no and %)

Possible modalities of exposure to pets	No (%)	DOG, no (%)	CAT, no (%)	DOG/CAT, no (%)
Previous ownership	150 (20.75 %)	65 (8.9)	62 (8.5)	23 (3.2)
Direct domestic contact	213 (29.46 %)	104 (14.3)	88 (12.1)	21 (2.9)
Direct contact elsewhere	146 (20.19 %)	50 (6.9)	57 (7.9)	39 (5.4)
Indirect contact	103 (14.25 %)	23 (3.2)	46 (6.3)	34 (4.7)
No apparent contact	111 (15.35 %)	12 (1.7)	16 (2.2)	83 (11.5)

**Table 3:** Triggering of respiratory symptoms after exposure to pet allergens in 723 pet-sensitized patients (no and %)

	Type of response	Total, no (%)	DOG, no (%)	CAT, no (%)	DOG/CAT, no (%)
Are allergic respiratory symptoms triggered by direct pet contact?	Positive	375 (51.8)	56 (7.8)	259 (35.8)	60 (8.3)
	Negative	10 (1.3)	5 (0.6)	3 (0.4)	2 (0.3)
	No response	338 (46.7)	(*) All pets		
		Patients no (%)			
		723 (100)			

(*) No response regards both pets without distinction

## Discussion and conclusions

Common pet ownership with a stable presence of the animal indoors is usually considered the main index of exposure to cat/dog, with the consequent risk of inducing allergic sensitization. “Are there animals at home?” is the common query administered by doctors to patients in order to collect information on anamnesis during epidemiological studies on the relationship between exposure to pets and development of allergic sensitization (e.g. during the first phase of life to evaluate a “protective effect” of early exposure to cat/dog). The same query is commonly used also in clinical practice to establish the clinical significance of a SPT positivity to cat/dog allergens and, thus, to manage the sensitized patients (pet-avoidance measures, allergen immunotherapy, pharmacological treatment of respiratory symptoms etc.). This commonly used question should not be considered the main factor of exposure to pet allergens and, consequently, the main risk factor for allergic sensitization either in clinical practice and large epidemiological studies [12, 20, 21]. In fact, Fig. 1 shows that only the condition b is reported usually in the questionnaires utilized for large epidemiological studies as well as in clinical practice for collecting data on anamnesis. In the conditions a, c and d the presence of a pet at home should be considered “formally negative” in the questionnaires or anamnestic report, but the level of direct/indirect exposure to pet allergens could be significant [4–7]. Only the condition e should be considered at the lower risk of pet allergen exposure after having excluded any direct/indirect contact with pets. Therefore, the simple answer “yes or no” on the question regarding the presence of pet at home can lead to misleading interpretation of the clinical significance of positive SPTs as well as the real risk of exposure to allergens of dog/cat in epidemiological studies. Consequently, we have previously suggested a new, more realistic, classification of modalities of exposure to pet allergens in “real life” based on the five possible conditions reported in Fig. 1. We have used this classification of exposure either for common pets and large animal such as horse, for which we have provided some specific modifications [10, 21–27]. To the best of our knowledge, this is the first study on the application of these new queries on the modality of exposure to pet allergens. As shown by Table 2, only a limited amount of patients sensitized to pets should be classified as “exposed to animals”, whereas the majority of patients should be classified as “not exposed” as a consequence of usual query “are there animals at home?. On the other hand and in agreement with our classification, a high percentage of formally “not-exposed” subjects were “really exposed” to pets. As a consequence of the present classification, only few patients were really “not-exposed” because no apparent direct/indirect exposure to pets or pet-derived materials. Another important finding of our study is that only half of our pet-sensitized individuals reported a clinically relevant symptoms worsening as a consequence of a close contact with pets, especially with cats. If we consider the modality of exposure, it is likely that these individuals belong to the groups directly exposed to pets at home or elsewhere [28]. These findings confirm that in already pet-sensitized patients a direct and prolonged exposure to animals may represent a relevant risk factor for exacerbations of respiratory symptoms [29]. It is important to note that 338 individuals (46.7%) failed to respond presumably because the symptoms were considered negligible, or not related with the contact of animals.

This is a possible limitation of this study. Other limitations are the lack of data on the presence of pet allergens at home for the reasons previously reported, and the lack of data on the general population regarding the exposure to pets in the first years of life.

**Fig. 2:**
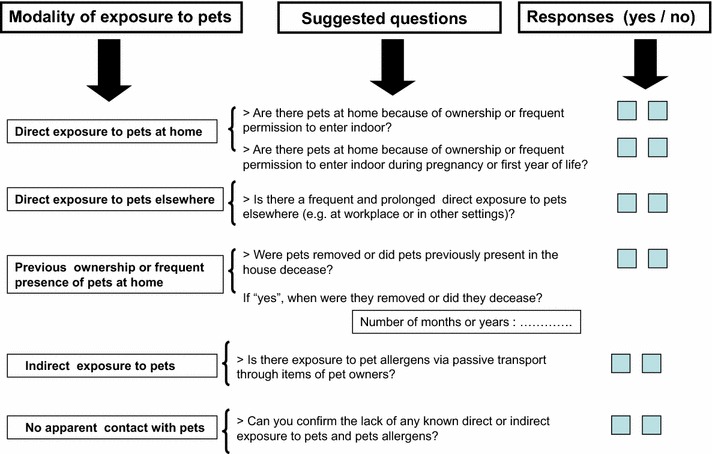
Suggested items to assess exposure to pet allergens in epidemiological studies and in clinical practice (Modified from [14])

In conclusion, this study suggests that our novel classification could be of particular importance to correctly evaluate the modality of pet exposure at home in the countries characterized by a high frequency of pet ownership. It is likely that, in these countries, the “average amount” of pet allergens indoors could be high (or very high in some particular conditions) also in the absence of a pet at home. The magnitude of exposure to pet allergens at home is not exclusively related to pet ownership/presence of a pet indoors, but can be also relevant without a pet living with the inhabitants. In addition, we have previously demonstrated, by using in vivo [30] and in vitro [31] methods, that allergic sensitization to common pets significantly increases the risk of developing sensitization to other furry animals, likely for cross-sensitization mechanism involving albumins and lipocalins. These considerations should be taken into account during the planning of epidemiological studies on the relationship between exposure to pet and development of allergic sensitization to pet allergens. In clinical practice, a real assessment of the risk and clinical significance of allergic sensitization to pet allergens is crucial for the management of patients (pet-avoidance measures, allergen immunotherapy, pharmacological treatment of respiratory symptoms etc.). In this context we have suggested few and well-defined questions to assess pet exposure in “real life” (Fig. 2) [14]. Finally, we believe that the topic of animal allergy is very important for both clinical and emotional implications in pet-owner patients, and especially in children. The love for animals in general and for pets in particular is increasing world-wide, so we wish to underline the necessity of an adequate assessment of risk factors for allergic sensitization, and possible prevention strategies by using a more realistic evaluation of possible modalities of exposure.
